# 

**Published:** 1800-06

**Authors:** 


### NEW MEDICAL PUBLICATIONS IN GERMANY.

*Hundbuch der Practiſchen Medicin*; i. e. Manual of Practical Medicine. By. J. ARNEMAN, Prof. of Medicine at the Univerſity of Göttingen, 8vo. 296 pp. 1800. Göttingen, Rugsrecht.

*Syſtem der Practiſchen Heilkunde*; i. e. Syſtem of the Practice of Phyſick, &c. By. Dr. HUFELAND, Prof. of Jena, 8vo. 1800. Jena, Fromman.

J. S. PALLAS *Species Aſtragalorum Deſcriptae et cum Iconibus Coloralis*; illuſtratae cum Appendice, T. I. et II. fol. 1800. Lips. Martini.

*Verſuch einer Vollſtandigen Geſchichte der Hernu. Nervenlehre*; i. e. Eſſay of a Hiſtory of Neurology. By Prof. HARLES, of Erlangen, 8vo. 1800. Erlangen, Schubart.

---

### To CORRESPONDENTS.

*The queſtion propoſed by J. W. cannot be diſcuſſed or anſwered in any periodical work; but we believe that any reſpectable phyſician will give him a ſatisfactory anſwer.*

*An anonymous Correſpondent thinks the caſe of mortification of the bladder, related in our laſt, was a retroverſion of the uterus.*

*Communications are received from Meſſrs. Leeſon, Hill, Kelſon, Leeſe, Schaw, Cuſtance, and Mitchell, which ſhall be noticed in due courſe.*

### INDEX.

Page.

ACIDS acetic and acetous, Cits. Adet and Chaptal, on the difference between 172
Acid camphoric and camphire, obfervations on, by Bouillon La Grange 89
—— metallic, Cit. Vauquelin, on a new one called chrome 173
—— nitrous and opium, their effects in the cure of dyfentery 413
Acid, new one difcovered by Mr. Klaproth of Berlin 583
Æthiop's mineral, method of preparing it 269
Æther, nitric, on the preparation of it without fire 86
———, Lowitz on an eafy method of depriving it of its alcohol *ib.*
Affections chlorotic, Dr. Scott's communication relative to 107, 215
Agaric, on a poifonous fpecies of, gathered in the Park 41
Alcalis, Tromfdorf's method of rendering them cauftic 86
Alderfon, Mr. his cure of a lufus naturæ 402
Alibert, Cit. on the colic of Madrid 167
————, on the treatment of leprofy *ib.*
————, on medicinal fubftances applied in frictions *ib.*
Aliments produced by different claffes of the animal kingdom, remarks on 153 249
Allan, on the effects of falt of tartar in puerperal fevers 80, 264
Alps, on the origin of fome rocks in them 375
Alumine, account of the fluat of 272
Ammonia, fulphat of, obfervations on 169
Amputation, an uncommon occurrence after 4
Anatomy, hiftory of the principal difcoveries in 72, 156, 256
————, Soemering's, Macartney's tranflaion of it announced 180
Andre, his letter to Dr. Pearfon on the report of perfons having died under the cow-pock inoculation 411
Aneurifm, Reeve on operation for the 531
Animals, Carlifle's difcovery of a peculiar arrangement in their arteries 490
Animal food, remarks on, and its influence on the body 65
——— Life, Dr. Buniva's obfervations on the doctrine of 272
Animation fufpended, Williams's obfervations on 222

Page.

Antoine Alexis, account of his hofpital for patients in hydrophobia 489
Apparatus employed in relieving odontalgia, explanation of 403
Arm, amputation of, obfervations upon it by Cit. Sabatier 368
Arfenic, teft of its being contained in fulphur 269
Art curative, Cit. Hallé on the hiftory of it 172
Atrophy, idiopathic fimple, Cit. Hallé on a cafe of 375
Azote oxydated, phenomenon in 91
Bailey, on the medicinal effects of digitalis 127
Bany, Dr. on vaccine 503
Bark, Goodwin on the ufe of 442
Bartley, Mr. on the pernicious effects of quackery 433
Barytes, muriat of, obfervations on it, by Dr. Richter 170
——— muriated, Mr. Juch's new method of preparing 581
Batty, Dr. his lectures announced 377, 588
Beddoes's, Dr. remarks on a German review of his effay on confumption 274
———— his letter to Dr. Bradley 486
————, his lectures on the laws of animal nature, announced 488
Beer, its antifeptic property 90
Beet-root, from whence Mr. Achard obtained fugar, letter to Dr. Bradley concerning it 8
Bell, on burns and fcalds 206, 296
——, his treatife on wounds tranflated into German 377
Beryl of Siberia, difcovery of a new earth in it 587
Biftoire caché, Carlifle's obfervations on it, and engraving of that inftrument as ufed in France by M. Deffault 193
————, reference to the plate 194
————, Watts's defcription of an improved one 195
————, explanation of the drawing of an improved one 197
Bitter extracts, Cit. Demachy on 581
Blackburn, his recipe for forming an agreeable and ufeful mixture with fpermaceti 515
Blackburn, Dr. on light 501
Bladder, account of a mortification of it, by Dray 456
Blair, Mr. his letter relative to the effect of living with cows in phthifis pulmonalis 14
Blane, Dr. his account of an uncommonly carnivorous man 209

Blood, on the circulation of, in certain animals 376
Bodies compound, Lamark on the effential particles of 89
——, the importance of opening after death 17
—— odoriferous, Brongniart on the effluvia of 89
Books Medical, Critical Retrospect of 93
Dr. Blane's Obfervations on the Difeafes of Seamen, 94. Pharmacopœa Boruffica, 93. Dr. Gibbons's Medical Cafes and Remarks, 94. Dr. Pearfon's Obfervations on Bilious Fevers, *ib.* Prof. Schmid's and Saumarez's New and Philofophic Syftem of Phyfiology, 181. Dr. Rufh's Lectures on Animal Life, 184. Prof. Göettling's Manual of the Theory and Practice of Chemiftry, 188. Henry's General View of Chemiftry, 189. Blair's fecond part of an Effay on Nitrous Acid, 190. Earle on the Effects of Fire, 191. Blair's fecond part of an Effay on Nitrous Acid, 275. Dr. Haygarth on Fictitious Tractors, 277. Dr. Cappel on Pneumonia Typhoide, 279. Prof. Schmid's Philofophic Syftem of Phyfiology, 280. Dr. Rufh's Lectures upon Animal Life, 283. New Effays by the Natural Hiftory Society at Berlin, vol. II. 284. Dr. Fothergill on the Prefervation of Shipwrecked Mariners, 285. Dr. Hooper's Anatomift's Vade Mecum, 286. Parkinfon's Villager's Friend and Phyfician, 287. Dr. Moffman on Scrophula, Glandular Confumption, &c. *ib.* Dr. Gren's Principles of Modern Chemiftry, 378. Heron's Elements of Chemiftry, 379. Dr. Jenner's Continuation on the Variolæ Vaccinæ, 381. Lipfcombe on the Hiftory and Caufe of Afthma, 383. Parkinfon's Chemical Pocket Book, *ib.* Anftruther on Heat, Electricity, and Light, 384. Dr. Sherer on Gafeous Bodies, *ib.* Dr. Hull's Elements of Botany, *ib.* Schelling's Outlines of Natural Philofophy, *ib.* Dr. Monro on the Burfæ Mucofæ, 386. Nayler on the Treatment of Ulcers, 387. Eddy on Ruptures, *ib.* Dr. Batfch's Botany for Ladies, &c. Dr. Kentifh's Letters to Cit. Baudelocque, 388 Riegel's Philofophy of Animals, *ib.* Prof. Vrolick's Oration on Vital Powers, 389. Dr. Parry on Syncope Anginofa, *ibid.* Burns on the Anatomy of the Gravid Uterus, 478. Dr. Rowley's Cogent Reafons why aftringent Injections, &c. fhould be banifhed from Practice, 480. Drs. Duncan's Annals of Medicine for the Year 1799, 482. Letter to Thomas Keate, Efq. Surgeon General to the Army, 486. Drs. Duncans' Annals of Medicine for the Year 1799, 552. Webfter's Hiftory of Epidemic Difeafes, 559. A Medical Practitioner on the good Effects of Yeaft and Nitric Acid in infectious Fevers, 561. Kirwan's Effay on the Analyfis of Mineral Waters, 562. Medical Facts and Obfervations, 563. Pearfon's Obfervations on the Effects of various Articles in the Materia Medica in the Cure of the Lues Venerea, 564 Valtelen's Pharmacologia Univerfa, 576. Dr. Acharius Lichenographiæ Suevicae Prodromia, 578. Dr. Auguftin's Syftem of Medicine, *ib.* Dr. Careno's Tranflation of Jenner's Enquiry, 579
Bottentuit's, Cit. reflections on Bourguet's obfervations on a cafe of paralyfis of the extenfor mufcles of the hands and fingers 253
Bougies armed with cauftic, Carlifle on the indifcriminate ufe of 292
Bouillon Lagrange, on camphire and camphoric acid, and on the analyfis of cork 89
Bouvier, on the oil of juniper 90
Bradley, Dr. his fpring lectures announced 92, 588
Brain, puriform deftruction of it without any apparent caufe 70
Brande, Mr. on a poifonous fpecies of agaric gathered in the Park 41
Bronchotomy, Bryer on a cafe of 228
Brongniart, on the effluvia of odoriferous bodies 89
——, on the affinities of oxygen with carbon 374
——, on the characters in defcribing minerals 375
——, on the origin of fome rocks in the Alps *ib.*
Brown, Dr. his obfervations on odontalgia 403
——, on the ufe of yeaft in typhus 533
Brown's obfervations on Zoonomia, S. N's anfwer to J. Y. refpecting 139
Brugnatelli on concentrating lemon-juice 171
Brunonian fyftem, Mr. Franks's obfervations refpecting it 47
Bryer, on a cafe of Bronchotomy 228

Buniva, on the doctrine of animal life 227

——, his experiments with injections into the blood vessels 587

Burns and scalds, Bell upon 206, 296

——, the use of spirit. terebinth. in 262

Carbon, on its affinities with oxygen 374

Carlisle, his discovery of a peculiar arrangement in the arteries of animals 490

——, on the indiscriminate use of bougies armed with caustic 289

Carson, Dr. on the various uses of digitalis 513

Cataract, Reimarus on the operation for 367

Catalepsy, Mr. Ring's case of 402

Cavander's substitute for coffee 270

Carnivorous man, Dr. Johnston's account of an uncommon one 209

Chambon, his work on the diseases of women and children 489

Chapman, on the depression of the skull 444

Christie, on Puerperal Convulsions 449

Clarke, on mixtures with spermaceti 263

Coffee, the use of, prohibited in Sweden 270

——, Cavander's substitute for *ibid.*

——, Mitsch.ng's substitute for 271

——, Piepenbring's substitute for *ibid.*

Colic of Madrid, Miller on it 167

Colour, preservation of, in the petals of flowers, by Cit. Haüy 91

Concretion, gouty, particulars respecting one in a man's finger 174

Consumption, remarks on a German Review of Dr. Beddoes essay on 274

Contagion, on the means of destroying it, by Dr. Trotter 245

Convulsions, puerperal, Christie on 449

Cow-pock inoculation, Dr. Pearson's letter concerning a report of two persons having died under it 411

Cow-pox, Dr. Denman, on the advantage likely to result from the inoculation for it 292

——, Grose, on its origin 294

Crichton, Dr. his lectures announced 179

Croup, Mr. Purton on it, in Answer to Dr Hu gan 240

Croup, Messrs. Huggan and Field's method of curing it 56

Crystals formed by mixing oil of rosemary with a solution of gold 88

Cuvier, Cit. on the circulation of the blood in certain animals 376

——, on the nutrition of insects *ib.*

Davies, on the adhesion of the placenta 6, 333

——, Mr. in reply to Dr. Squire, relative to delivery of the placenta 536

Davies, Mr. Peck's Observations on his case of adhesion of the placenta 516

Deaths in Bills of Mortality for the last three months 408

Delirium, Dr. Shapter on a case of 238

Delivery, premature, case of, communicated by Dr. Denman 3

Denman, Dr. his account of a case of polypus uteri 1

——, his mode of relating cases recommended for imitation *ibid.*

——, on a case of premature delivery 3

——, on the advantages by inoculating for the cow-pox 292

Demachy, Cit. on bitter extracts 581

De Launay, Cit. on an easier method of making mercurial ointments *ib.*

Deyeux, on the effects of salt of tartar in puerperal fevers 165

Digitalis, observations on 126, 127, 128, 305, 307, 311, 313, 515

——, Dr. Maclean in Answer to Dr. Drake, concerning it 150

——, Dr. Drake, on the use of 305

——, Dr. Sherwin on 307

—— Dr. Carson on 513

Diseases, account of, in an eastern district, from the 20th of November to the 20th of December, 1799 15

Ditto, from the 20th of December, 1799, to the 20th of January, 1800 109

——, from the 20th of January to the 20th of February, 1800 207

——, from the 20th of Feb. to the 20th of March, 1800 302

——, from the 20th of March to the 20th of April, 1800 407

——, from the 20th of April to the 20th of May 504

——, in London, Dr. Willan's observations respecting 297

Diseases, account of, at the west-end of the town, from November the 10th to December the 20th following 14

——, in the Westminster Hospital, 16, 112, 208, 303, 408, 504

Diseases, monthly report of at the Finsbury Dispensary 391, 409, 506

Diseases and casualties of the year 1799 111

Disease, venereal, on the treatment of, by Dr. Vage 465

Diseases of women and children, Chambon's work on 489

Disease venereal, Pearson's new work on, continued 274

Distillation, account of Suboff's improvement in 272

Docker, Mr. on opiate frictions 102

Dray, Mr. on Dr. Huggan's opinions 326

Dray's account of a mortification of the bladder 456

Dunning, Mr. on strangulated hernia 330

Dyce, Dr. on a case of polydipsia 509

Dysentery, Dr. Whyte, on the treatment of 231

———, Mr. Hope's observations on the effects of nitrous acid and opium, in its cure 413

Dyspnœa, Mr. Lipscomb on 520

Eacard, his method of preparing a new tincture of opium 582

Earth, a new one discovered in the beryl of Siberia, by Cit. Vauquelin 587

Emerald, its analysis 173

Enemata, Blythe on the use of 229

Epidemic at Strasburgh 377

Establishments, new, in Saxony, account of *ib.*

Evans, Dr. on the good effects of alkaline salts in counteracting the poison of corrosive sublimate 535

———, on compouud fracture 549

Faivre, on opium and mercury in lues 168

Fœtus, case of extraordinary malformation in (with plate) 397

Fevers puerperal, effects of salt of tartar in 80, 264

Fever from venereal poisons, Dr. Haygarth's remarks on 198

Fevers, Prof. Reiche's new theory of 586

Field, his method of curing the croup 56

Finch, Rev. Mr. on the vaccine inoculation 415

Finsbury Dispensary, monthly report of diseases at 391, 409, 506

Fistula Lachrymalis, Pajot des Charmes on alkaline substances in the treatment of 168

Fistula in ano, Whately on 224

———, Mr. Whately's description of a new instrument for performing the operation for 493

Fourcroy, on the treatment of leprosy 167

———, on human urine 174

Fœtus, evolution of it effected by the action of the uterus 5

Fowler, Mr. his observations on the destruction of the teeth 55

Frictions, medicinal substances applied in 167

Fumigation nitrous, Dr. Trotter's observations on 321

———, Dr. Yeats on 429

Girod Chantran, on the antiseptic property of beer 90

Goodwin, on the use of bark 442

Graff, his experiments to obtain sugar from different vegetable substances 582

Grandechamps, Cit. his two instances of preternatural ossification 587

Grose, on the medicinal use of yeast 105

Guinot, on the effects of salt of tartar in puerperal fevers 165, 264, 363

Gun-shot wounds, continuation of Christie's observations on 141

Hæmoptoe, Mr. Hull's account of a case of 424

Hæmorrhoids, Custance on 334

Hagstrom, Dr. on chronic dysphagia 366

Hahnemanns, Dr. liquor vini probatorius fortior 171

Hair, Lanoix on cutting it after acute diseases 367

Hallé, on the treatment of leprosy 167

———, Cit. on a case of simple idiopathic atrophy 375

Harrington, Dr. on the medicinal properties of digitalis 202

———, his cases of anarsarca of the lungs *ib.*

———, his cases of hydrops pericardi 203

———, his cases of hydrops thoracis 204

———, his cases of hydrops ascites 205

Haygarth, on fever from venereal poisons 198

Head, its injuries, observations on six cases of 30

Hewitt, on a calculus of the tonsils 446

Hermbstädt, Dr. on the chemical analysis of vegetables 370

———, Prof. his discovery of decomposing sea salt 376

Hints and Improvements in Medicine, Surgery, and Pharmacy 262, 363

Hope, on the effects of nitrous acid and opium in the cure of dysentery 413

Hufeland, Prof. on the sulphat of ammonia 169

———, Dr. on the seed of the thorn-apple as a narcotic remedy 488

Huggan, his method of curing the croup 56

———, on Venæsection, &c 538

Hull, his account of a case of hæmoptoe 424

Huggan on venæsection, Urvin's strictures on 226

Hydrocephalus, Mr. Reeve on a successful case of 61

——— internus, Mr. White on 113, 325,

———, Mr. Shaw on a case of 517

Hydrophobia, account of an hospital for patients in 489

Hydrops pericardi, case of 203

Hydrops thoracis 204

——— ascites 205

Inoculation vaccine, Stewart's observations on 234

Inoculation Vaccine, obfervations on, communicated by Dr. Remmet 237
————, Dr. Huggan's remarks on 243, 344
————, Rev. Mr. Fofbrooke on, in reply to Dr. Pearfon 247
————, at Manchefter 274
————, Shorter's obfervations on 348
————, Dr. Jenner on *ib.*
————, Rev. Mr. Finch on 415
————, Mr. Dunning on 436
————, in Hanover, Stromeyer's letter concerning 471
————, Dr. Trotter on 524
————, Dr. Jenner's anfwer to the queries refpecting it 502
Intelligence, Medical and Phyfical, v. Medical and Phyfical Intelligence
————, domeftic 92, 175
————, mifcellaneous 270, 377, 487
Ink for writing, Ribaucourt's receipt for making an excellent one 175
Jenner, Dr. on the eruptions which fometimes appear in the inoculated vaccine difeafe 101
Jenner, Dr. his anfwers to the queries on vaccine inoculation 502
Johnfton, Dr. his account of an uncommon carnivorous man 209
Juniper, oil of, obfervations on, by Bouvier 90
Juch, his new method of preparing the muriated barytes 581
Kaftelyn, on vitrolated kali 169
————, on the preparation of magnefia 170
Kentifh, Dr. on the ufe of fpirit. terebinth. in burns and fcalds 262
Kinglake, Dr. on the medicinal effects of digitalis 120
Klaproth of Berlin, his difcovery of a new acid 583
Lacroix, on the analyfis of fnow-water 90
Lamark, on the effential particles of compound bodies 89
Lanoix, on cutting the hair after acute difeafes 367
Larrey, on the diforganization of feveral vifcera 168
————, on an imperforate vagina *ib.*
Lartique Cit. his new method of preparing tartar emetic 582
Lectures announced, Dr. Bradley's 92, 588
————, Dr. Pearfon's 179
————, Dr. Crichton's 179
————, Dr. Batty's 180, 588
————, Mr. Fox's 274
Lefebure, on dangerous metals for culinary utenfils 90
Lemon-juice, Dr. Brugnatelli on concentrating it 171
Leprofy, treatment of 167
Light, its influence on metals 91
Light, Dr. Blackburn on 501
Lipfcomb, on dyfpnœa 520
Liquor vini probatorius fortior of Dr. Hahnemann 171
———— anod. vegetab. et naphta aceti 267
London, difeafes in, obfervations refpecting 297
Lucius, M. on the influence of light on metals 91
Lues, Faivre on opium and mercury in 168
Lufus Naturæ, cafe of in a child 137
————, Mr. Alderfon's cafe of one 402
Maclean, Dr. on Digitalis 150
Mackie, Dr. his account of a cafe of offified uterus 422
Magennis, on the medicinal effects of digitalis 128
Magnefia, Kafteleyn on the preparation of 170
————, on the preparation of, by Prof. Tromfdorf and Cit. Van Mons 87
Malformation, Dr. Vaughan on 495
————, Mr. Pole's appendix to his cafe of 497
Mansfield, Mr. his account of the converfion of a large part of a female body into fatty matter 19
Martineau, Mr. on the affufion of cold water in cafes of fever 51
Mayow's difcoveries, Dr. Clarke on 335
Medical Foreign Literature 568
Medical and phyfical intelligence 88, 172, 585
Medical publications, new, in December 95
————, in January 192
————, in Germany 96, 192, 492, 588
————, in February 287
————, in France ibid.
Medical publications, new, in March 395
————, in France *ibid.*
————, in April 491
Medicine, hints and improvements in 80
Mitfching's fubftitute for coffee 271
Monftrofity, cafe of 138
Moore, on ulceration in the ftomach 511
Moffman, Dr. on digitalis 311
Neffe Hill, his obfervations on the inconvenience of healing wounds by the firft intention 12
Nymphæa nelumbo, an eaftern cofmetic, Pallas's account of 273
Odontalgia, obfervations on, by Dr. Brown 403
————, explanation of an apparatus employed in relieving it 403

Ointment and plaifters, new method of preparing 170
Ointment mercurial, Cit. Delaunay on an eafier method of making 581
Opiate frictions, Mr. Docker's obfervations on 102
Opium and nitrous acid, their effects in the cure of dyfentery 413
Opii tinctura, Mr. Eacard's new method of preparing it 582
Opium falivation, from 586
Offification preternatural, two curious inftances of, by Cit. Grandchamp 587
Oxygen, Cit Fourcroy's new method of combining with fuet 580
——, Cit Brongniart on its affinities with Carbon 374
Pajot des Charmes, on alkaline fubftances in the treatment of fiftula 168
Paralyfis of the extenfor mufcles of the hand and fingers, obfervations on a cafe of, by Cit. Bourguet 253
Pallas's account of Subof's improvement in diftillation 272
——, account of the nymphæa nelumbo, an eaftern cofmetic 273
Paris, Memoirs of the Society of Natural Hiftory at, announced 272
Parturition, difficult cafe of, with obfervations by Dr. Clarke 44
Payne on a new poultice 164
Pearfon, Dr. his letter concerning a report of two perfons having died under the cow-pock inoculation 411
——, Andre's anfwer to his letter on the above report 412
——, on the vaccine pock inoculat on 92
——, Mr. annunication of his fpring courfe of lectures ib.
——, Dr. on the eruptions refembling the fmall-pox, which fometimes appear in the inoculated vaccine difeafe 97
——, Mr. his lectures announced 179
——, his new work on the venereal difeafe, announced 274
——, Dr. on the prefent ftate of the evidence refpecting vaccine inoculation 399
Peck, on the expediency of an early delivery of the placenta 221
——, his obfervations on a cafe of adhefion of the placenta 516
——, in reply to Dr. Squire, relative to an early delivery of the placenta 529
Penkevil, Mr. on digitalis 315
Pharmacy, hints and improvements in 80
Pharmaceutical proceffes 85, 169, 267, 580
Phofphorus, medicinal 270
Piepenbring's fubftitute for coffee 271
—— for tea ib.
Phthifis pulmonalis, the effects of living with cows in this diforder 14
Phymofis, on the treatment of 162
Placenta, Davies on the adhefion of 335
——, Wagftaffe on an adhefion of it 421
——, Scott, Dr. on an adhefion of it 447
——, Dr. Squire on the extraction of it 459
——, adhefion of it, obfervations upon Mr. Davies's cafe by Mr. Peck 516
——, on the expediency of an early delivery of it 221
——, an adhefion of after delivery 6
Plaifters and ointments, new method of preparing 170
Plants, M. Rafn on the vegetation of 175
Poifon, Venereal, Dr. Haygarth on fever from 198
Pole's appendix to his cafe of malformation 495
Polypus uteri, cafe of, communicated by Dr. Denman 1
Polydipfia, cafe of by Dr. Dyce 509
Poultice, obfervations on a new one 164
Pox, fwine, obfervations upon it 219
Proceffes, pharmaceutical, fee pharmacentical proceffes
Punch, obfervations upon, as a fubftitute for wine in typhus 217
Purton, Mr. his cafe of difficult parturition, with Dr. Clarke's obfervations 44
Quackery, the pernicious effects of 433
Quackery, Mr. Urvins on 523
Ribaucourt's receipt for an excellent writing ink 175
Reich, Prof. his arcanum in cafes of fever 586
——, on a new method of combining oxygen with fuet 580
Reimarus, Dr. on the operation for the cataraact 367
Remmet, Dr. his letter on hernia 332
Reeve, on the operation for aneurifm 531
——, Mr. on a fuccefsful cafe of hydrocephalus 61
Reid, Dr. his obfervations on the treatment of General Wafhington in his laft illnefs 473
Ring, his cafe of catalepfy 402
Rowlands, Mr. on an uncommon occurrence after amputation 4
——, on the evolution of the fœtus, effected by the action of the uterus 5
Ruby, its analyfis 173

Salt, on a new species of, and its use in several diseases 91

—— of tartar, its use in puerperal fevers 80, 264

Sea Salt, Prof. Hermbstädt's discovery for decomposing it 376

Scherer, on a phenomenon in oxydated azote 91

Scott, Dr. on adhesion of the placenta 447

Shaw, on a case of hydrocephalus 517

Shapter, Dr. on a case of delirium 238

Sherf, Dr. notice of his Journal of Medicine, Jurisprudence, and Police 274

Sherwen, Dr. on digitalis 307

Simmons, Dr. his 8th vol. of Medical Facts and Observations, announced 92

Skull, Chapman on the depression of it 444

Smith, Dr. his Flora Britannica announced 489

Snow water, La Croix on its analysis 90

Soap, mercurial 270, 582

Soda, Lelievre on its extraction from marine kali 90

——, Van Mons on its separation from marine salt 169

Spermaceti, on its unsuccessful combination with water in mixtures 515

——, improved formula for making a mixture by combining it with water *ib.*

——, Clarke on mixtures with 263

Spina bifida, Mr. Thackery's observations on 104

Spirit. terebinth, its use in burns and scalds 262

Sprengel, on the principal discoveries in anatomy 156, 256, 321

Spry, Mr. on the importance of opening bodies after death 17

Stomach, Moore on ulcerations in it 511

Stewart, on vaccine inoculation 234

Stockholm, account of patients in the Royal Hospital there 174

——, uncommon mortality in the house of education there 175

Stokes, Dr. his letter to Dr. Bradley concerning the beet-root 8

——, on Mayow's discoveries 335

Strangulated hernia, Dunning on 330

——, Dr. Remmett on 332

Substances alkaline, their use in the treatment of fistula lacrymalis 168

Sugar, Mr. Gräff's experiment to obtain it from different vegetable substances 582

Sulphur, liver of, recommended in some cases of struma 586

Teeth, on their destruction, by Mr. Fowler 55

Tartar salt, of, its effects in puerperal fevers 80, 264

Tartar emetic, new method of preparing it, by Cit. Lartique 581

Thackeray, Mr. on a case of spina bifida 104

Tromsdorff, on the sulphat of ammonia 169

Trotter, Dr. on the means of destroying contagion 245

——, on vaccine inoculation 524

——, in reply to Dr. Yeats 526

Tungsten, Mr. Kirchhoff's method of decomposing its oxyd, via humida 87

Vaccine disease, observations on the eruptions resembling the small-pox which sometimes appear in this disorder 97

Vaccine inoculation, see inoculation vaccine

——, Dr. Pearson on the present state of the evidence respecting 399

——, Stromeyer's letter concerning it in Hanover 471

—— pock, account of the institution for the inoculation of it 175

Vage, Dr. on the treatment of the venereal disease 465

Vagina, Larray on an imperforate one 168

Van Mons, on the separation of soda from marine salt 169

——, on an improved method of preparing plaisters and ointment 170

Vauquelin, Cit. his discovery of a new earth in the beryl of Siberia 587

Vaughan, Dr. on malformation 495

Variolæ vaccinæ, Dr. Jenner's treatises on 179

Vauquelin, Cit. on the extractive principle of vegetables 173

——, on a gouty concretion on the fingers of a man 174

——, on a new metallic acid called chrome 173

——, his analysis of the ruby *ib.*

—— emerald *ib.*

——, on chrystals formed by oil of rosemary and gold 88

Vegetables, on their extractive principle 173

——, Dr. Hermbstädt on the chemical analysis of 370

Venæsection, Huggan on Urwin's strictures on 226

Viscera, on the disorganization of several 168

Viator, on punch as a substitute for wine in typhus 217

Virey, his remarks on animal aliments 6[illegible], 153, 249

Urvins, Mr. on quackery 523

Ulcerated legs, on the neceffity of erecting an hofpital for the cure of 135
Urine human, on a chemical and medical hifto y of 174
Uterus offified, Dr. Mackie's account of one 422
Wagftaffe, on an adhefion of the placenta 421
Wafhington General, Dr. Reid's obfervations on his medical treatment in his laft illnefs 473
———, Drs. Craig and Dick's report of his death 490
Water, on the prefervation of in long voyages by Vauquelin 90
Watt's defcription of an improved biftoire caché 195
Water, cold, on its affufion in fever 51
Weftminfter Hofpital, account of difeafes in 16, 112, 208, 303, 408, 505
White on hydrocephalus internus, 113, 325
Whyte, Dr. on the treatment of dyfentery 231
Whately, his defcription of a new inftrument for performing the operation of fiftula in ano 493
Willan, Dr his lift of difeafes at the weft-end of the town, from Dec. 20 to March 15 297
William's obfervations on fufpended animation 222
Willich, Dr. his addrefs to an anonymous reviewer 180
———, his lectures, 3d edition 377
Wounds, gun-fhot, A. B. on a cafe of 353
———, Bell's treatife on, tranflated into German 377
———, inconvenience of healing them by the firft intention 12
———, gun-fhot, continuation of Mr. Chriftie's obfervations on 34
Yeat's, Dr. on nitrous fumigati on 429
Yeaft, Grofe on the medicinal ufe of 105

### *References to the Plates.*

| Plate | | Page |
|---|---|---|
| Plate IV. | An Apparatus for applying Smoke to a decayed Tooth | 20 |
| | A double Intus-Susceptio which happened in a Child, aged 6 months | 403 |
| V. | An Improvement of the *Bistoire Caché* | 193 |
| | Mr. Robert Watts's new Inftrument for Lithotomy | 195 |
| VI. | An extraordinary Malformation of a Fœtus | 397 |
| VII. | A new Inftrument for performing the Operation of Fiftula in Ano | 493 |
| | A Drawing of a Compound Fracture of the Leg, with a confiderable Protrufion of the Tibia | 549 |

Printed by William Thorne, Red Lion Court, Fleet Street.

